# Effect of Type-I Interferon on Retroviruses

**DOI:** 10.3390/v1030545

**Published:** 2009-10-27

**Authors:** Esperanza Gómez-Lucía, Victorio M. Collado, Guadalupe Miró, Ana Doménech

**Affiliations:** Departamento de Sanidad Animal, Facultad Veterinaria, Universidad Complutense, 28040 Madrid, Spain; E-mails: vmcollado@vet.ucm.es (V.M.C.); gmiro@vet.ucm.es (G.M.); domenech@vet.ucm.es (A.D.)

**Keywords:** Type-I interferons, retrovirus, HIV-1, HTLV-I, FeLV, MuLV, ISRE

## Abstract

Type-I interferons (IFN-I) play an important role in the innate immune response to several retroviruses. They seem to be effective in controlling the *in vivo* infection, though many of the clinical signs of retroviral infection may be due to their continual presence which over-stimulates the immune system and activates apoptosis. IFN-I not only affect the immune system, but also operate directly on virus replication. Most data suggest that the *in vitro* treatment with IFN-I of retrovirus infected cells inhibits the final stages of virogenesis, avoiding the correct assembly of viral particles and their budding, even though the mechanism is not well understood. However, in some retroviruses IFN-I may also act at a previous stage as some retroviral LTRs posses sequences homologous to the IFN-stimulated response element (ISRE). When stimulated, ISREs control viral transcription. HIV-1 displays several mechanisms for evading IFN-I, such as through Tat and Nef. Besides IFN-α and IFN-β, some other type I IFN, such as IFN-τ and IFN-ω, have potent antiviral activity and are promising treatment drugs.

## 1. Retroviruses

Retroviruses are enveloped viruses, with single stranded RNA, and with an unusual replication strategy through a double stranded DNA intermediary. This process is accomplished thanks to the enzyme reverse transcriptase, which directs the synthesis of DNA. Once as DNA, the nucleic acid integrates in the genome of the host cell, where it behaves as another gene. Retroviral particles are surrounded by an envelope, which contains two types of viral glycoproteins: SU (surface) and TM (transmembrane); under the envelope the matrix proteins (MA) confer stability to the viral particle. The core or capsid is mostly formed by capsid proteins (CA); it lodges inside the RNA surrounded by the proteins of the nucleocapsid (NC), and the enzymatic proteins: protease (PR), retrotranscriptase or reverse transcriptase (RT) and integrase (IN). Some viruses also have the enzyme dUTPase (DU).

**Figure 1.**
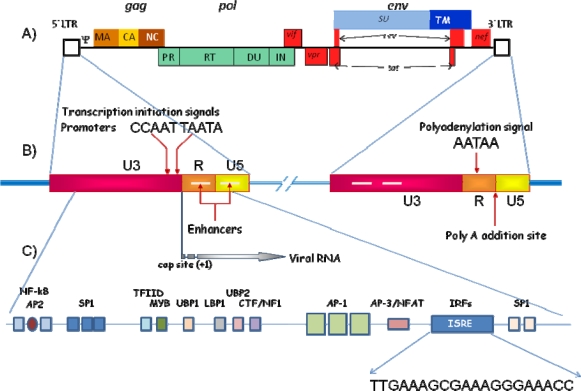
A) Schematic representation of the genomic structure of HIV-1, B) Blow-up of the two Long Terminal Repeats (*LTR*) which flank the proviral sequence, C) Some of the regulatory elements in the 5′ LTR, showing the sequence of the Interferon-Stimulated Response Elements (*ISRE*).

Viral particles have two identical copies of the ARN genome, which have a cap sequence in the 5′ end and a polyadenylated tract or poli(A) in the 3′ end, but do not function as mRNA. Four genes are necessary for infectious viruses: *gag*, *pro*, *pol* and *env* (Figure 1). Gene *gag* (group specific antigen) codes for the structural internal proteins of the virus (MA, CA, NC and other proteins specific to certain viruses with undetermined function). Enzymatic proteins necessary for viral replication are coded by genes *pro* (protease, PR). and *pol* (polymerase, IN, RT and, in some retroviruses, DU). In some retroviruses, *pro* is in the same ORF as either *gag* or *pol*. Gene *env* (envelope) codes for the proteins present in the envelope (SU and TM). Deltaretroviruses, such as human T-cell leukemia virus (HTLV) or bovine leukemia virus (BLV) have two additional genes, *tax* and *rex* [100], whereas lentiviruses posses, besides the mentioned genes, other genes (such as *tat, rev, vif*, *vpr*, and others) which encode for non-structural proteins relevant for the regulation of expression, viral replication, pathogenesis, etc [100].

Depending on the virus, virions attach to a number of different specific cellular receptors via their envelope glycoproteins. In most cases, the virus envelope and the cell membrane fuse, allowing the virion core to enter the cytoplasm; in fewer instances, entry involves receptor-mediated endocytosis. Decapsidation, mediated by membrane proteases or by lytic enzymes in the phagolysosomes, is almost immediate. Thanks to the RT, a dsDNA molecule is synthesized using the RNA as template. During this stage, the RT adds 300–1200 base pairs (bp) in each end of the nucleic acid, which become the structures known as Long Terminal Repeats (LTRs, see below). The new dsDNA molecule crosses the nuclear membrane, and integrates (by ways of the viral integrase) in the chromosomal DNA of the host cell. Initially it was believed that this process happened mostly at random, but nowadays it is recognized that different retroviruses display strong preferences in selecting their target sites in the genomes of their host cells [for a review, see 22]. For instance, murine leukemia virus (MuLV) prefers to integrate at transcription start sites, and human immunodeficiency virus (HIV)-1 displays a strong preference for highly active genes [130]. Many of the biological characteristics of retroviruses, such as the activation of oncogenes, or the possibility that retroviruses become endogenous and be transmitted through generations as Mendelian genes, derive from this stage. When the viral genome is integrated in the genome of the host cell it is termed “provirus”.

The provirus stays integrated in the host DNA during periods of time of variable length. At some point, mRNA is transcribed from the viral DNA, and once in the cytoplasm it is translated into the specific proteins of the virus. These proteins, along with viral RNA transcribed from the DNA, assemble to form new viral particles that are released from the cell budding from the plasma membrane.

### 1.1. Long Terminal Repeats (LTR)

The RNA genome of retroviral particles is flanked by two short repeated sequences (R), associated to the regions U5 and U3 in the 5′ and 3′ends, respectively. U5 and U3 are duplicated during the reverse transcription of the RNA genome into dsDNA, providing the provirus with a new terminal sequence U3-R-U5 flanking each end, which binds covalently to the host DNA (Figure 1). These new sequences are termed Long Terminal Repeats (LTRs); their size ranges between 300 and 1200 bp, depending on the retrovirus, and they are the control centers for gene expression, containing all of the requisite signals for this function: enhancers, promoters, transcription initiation (capping), transcription terminator and polyadenylation signal.

The transcription starts at the beginning of R, is capped and proceeds through U5 and the rest of the provirus, usually terminating by the addition of a poly A tract just after the R sequence in the 3′ LTR. The LTR contains two functional domains: a promoter region, and an enhancer region (Figure 1), which bind several protein factors (both general and tissue-specific). Enhancers are an important class of transcriptional regulatory elements, downstream from the transcription start site (*i.e. cis*-acting). Enhancers identified up-to-date show very little homology; they usually have a core domain, about 10–15 bp long, that binds a specific cellular factor (such as hormones, mitogens, cytokines, nutrients or toxins), which regulates their expression [53, 111]. Most of these elements have a positive effect on transcription, but some do have a negative effect. The different members of these families vary significantly in their relative abundance, in their functional activity and in their interactions with other additional proteins, generating a complex transcriptional enhancers’ network, which is considered crucial [34, 75, 134, 139]. Thus, the LTR sequences of retroviruses in general, but especially of lentiviruses, offer various possibilities for regulation. This, along with the activity of the accessory regulatory proteins, allows the virus to alter specific biochemical routes of the cell, and enhance viral survival and its spread within the host, permitting them even to invade cellular environments not permissible to other viruses, such as cells which are not dividing.

Some retroviruses contain additional enhancer sites, homologous to sequences detected in genes which are sensitive to the action of interferon (IFN), termed Interferon Stimulated Response Elements or ISREs (see below) (Figure 1). These sequences allow them to respond in a dose dependent manner to the presence of IFN, which thus directs transcription.

The 3′ LTR is not normally functional as a promoter, although it has exactly the same sequence arrangement as the 5′ LTR. The function of the 3′ LTR is to act in transcription termination and polyadenylation.

## 2. Interferon

Interferons (IFNs) are a multigene family of inducible cytokines, which regulate immunity in infectious diseases and tumours [11, 17]. IFNs are commonly grouped into two types [107]. Type I IFNs (IFN-I), which possess antiviral activity and are associated with the innate immunity, are also known as viral IFNs and include amongst others IFN-α (leukocyte IFN), IFN-β (fibroblast IFN), IFN-ω, and IFN-τ. Type II IFN (IFN-II), produced by activated T-cells and natural killer cells, is also known as immune IFN (IFN-γ) [16]. Type I IFNs induce antiproliferative and antiviral responses, and not only play an important role in the innate immune response, but also influence the generation of the adaptive immune responses [7, 52, 117].

The induction of type I IFNs is regulated by two signal transduction pathways, both of which are activated by viruses [6, 56, 143]: the classical and the Toll-like receptors (TLR) pathways.

**Figure 2.**
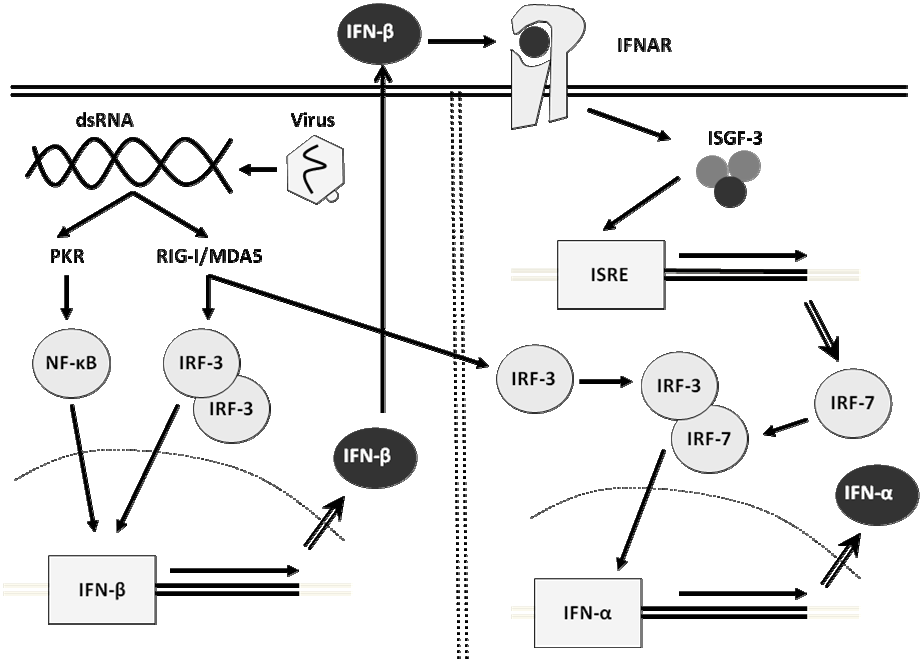
Simplified mechanism of regulation of the induction of the interferon genes in most cells. *PKR*, RNA dependent protein kinase; *IRF*, interferon regulatory factor; *ISGF*, IFN-stimulated gene factor; *IFNAR*, interferon alpha receptor; *ISRE*, interferon stimulated response elements. Ovals represent proteins, squares represent genes.

Most cells, including fibroblasts, hepatocytes, and conventional dendritic cells, use the so-called classical pathway. These cells have cytosolic receptors (CR) [6] that are able to recognize viral nucleic acids within the infected cells. CR includes the RIG-I-like RNA helicase receptor (RLH) family: retinoic acid inducible gene-I (RIG-I) and melanoma differentiation associated gene 5 (MDA5) [138]. The presence of viral dsRNA activates the routes of NF-κB and Interferon Regulatory Factor (IRF)-3, the main regulatory factors for IFN transcription. NF-κB and IRF-3 translocate to the nucleus and bind the promoter for the IFN-β gene, forming a complex named “enhanceasome”, which combines with other co-activators and RNA-polymerase, in order to transcribe IFN-β. Thus, cells secrete initially IFN-β. In the following amplification stage, IFN-β triggers the expression of IRF-7. This factor has been proposed as the main regulator of the expression of IFN-I genes [64], and, in collaboration with IRF-3, triggers the synthesis of IFN-α [56, 95] (Figure 2).

**Figure 3.**
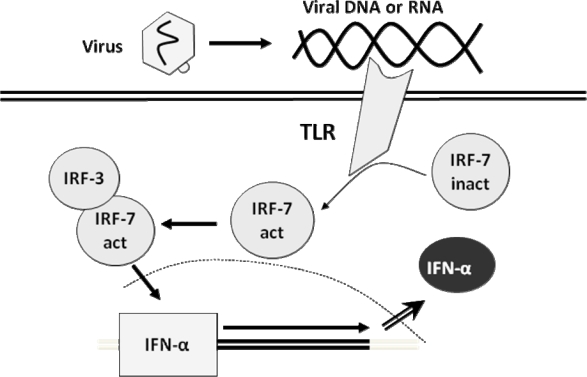
Simplified mechanism of regulation of the induction of the interferon genes in dendritic cells. *IRF*, interferon regulatory factor. Ovals represent proteins, squares represent genes.

Plasmacytoid dendritic cells (pDCs), also known as *precursors of type 2 dendritic cells* or *natural IFN-producing cells*, use the Toll-like receptors (TLR) pathway, expressed in the cell surface or in the endosomes, sensitive to viral DNA and RNA. The TLR signal activates IRF-7, which is constitutively expressed in these cells [50, 56, 58, 62, 65, 123], directly originating the secretion of high levels of IFN-α [31, 37, 49, 56] (Figure 3).

**Figure 4.**
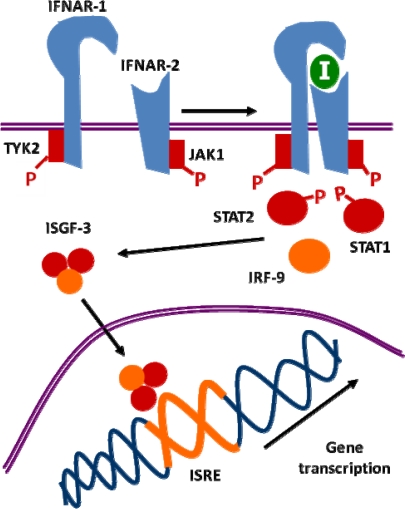
Induction mechanism of genes by type I interferons. *IFNAR*, IFN-α receptor; *IRF*, interferon regulatory factor; *ISGF*, IFN-stimulated gene factor; *ISRE*, interferon-stimulated response element; *TYK*, tyrosine kinase; *JAK*, Janus kinase; *STAT*, Signal Transduction and Activator of Transcription.

Once type I IFN is synthesized, it binds to the interferon alpha receptor (IFNAR), the specific receptor for IFN-I on the cell membrane, formed by two subunits: IFNAR-1 and IFNAR-2 (Figure 4). The binding of IFN-I to IFNAR-1 and 2 produces the heterodimerization of both subunits [10, 98, 135], which activates the tyrosine kinases TYK-2 and JAK-1. This phosphorylates a related transcription factor which is in the cytoplasm, and which is involved in the signal transduction of several molecules, termed STAT (Signal Transduction and Activator of Transcription) [12, 85]. Of the several STAT proteins, IFN-α or -β phosphorylate STAT-1 and STAT-2, which bind p48 (IRF-9), forming a trimer (IFN-stimulated gene factor-3, ISGF-3). ISGF-3 translocates to the nucleus and binds the so-called Interferon-Stimulated Response Elements (ISRE) in IFN-inducible genes (ISGs). As mentioned before, ISREs are *cis*-acting DNA regulatory sequences; they have a consensus sequence a/gNGAAANNGAAACT [142,146], very similar to the IRF elements (IRF-E, whose consensus sequence G(A)AAAg/ct/cGAAAg/ct/c is present within the promoters of IFN-I and of most ISGs) [96] (Figure 4). Antiviral activities associated with IFN-α include, among others, the induction of protein Mx GTPase, activation of protein kinase R (PKR) leading to mRNA translation, and activation of the 2′,5′-OAS/RNase L system, resulting in RNA degradation [54, 119].

IFN-I plays an important role in the defense against viral infections, both through IFN-induced proteins and enzymes mentioned above (innate response), as well as through its effect on the adaptive immune response, since the maturation of dendritic cells by IFN is important for the stimulation of T-cells [52, 117]. In addition, type I IFNs increase the expression of class I MHC molecules in all cells, contributing to the removal of the infected cells [7]. In HIV infections, results are controversial; some researchers have proposed that the effect of IFN may be beneficial, while other deleterious, since it may activate apoptosis of CD4+ T-cells, both non-infected and HIV-infected, through the expression of TNF-related apoptosis inducing ligand (TRAIL) and death receptor (DR) 5 in their membrane [60].

When IFN-I is administered to HIV-, simian immunodeficiency virus (SIV)-, or feline immunodeficiency virus (FIV)-infected individuals clinical signs are seen to improve [30, 33, 60, 106, 117], supporting the hypothesis that type I IFNs play important roles in the response to these retroviruses, as well as to other retroviruses, such as HTLV-I [46, 79, 117], BLV [78], feline leukemia virus (FeLV) [28, 149], or MuLV [2, 52].

These effects of IFN-I may be observed both *in vivo* and *in vitro*. In the following paragraphs evidence will be discussed on the observations on

- IFN-I levels in natural and experimental retroviral infection, and whether they are protective or deleterious, and association to specific syndromes, such as dementia
- Possible impairment of pDCs as the cause of altered IFN levels
- Effects of IFN on virogenesis
- Effects of IFN at the cellular level: induction of apoptosis in infected and non infected cells
- Effects of IFN at the molecular level: presence of ISREs in retroviral genome (LTRs)
- Mechanisms by which retroviruses may evade the effect of IFN-I

Most data originate from studies performed with IFN-α. However, due to their similarity, in the next sections either IFN-α or IFN-β are referred to as IFN-I. In the last section, some results obtained with other type I IFNs are reviewed.

## 3. Evidence of the Importance *in Vivo* of Type I Interferons in Retroviral Infections

### 3.1. Systemic IFN levels and their relationship with pathogenesis

Most of the data regarding the relevance of IFN-I on retroviruses stem from results on the infection with HIV. Several data suggest that *in vivo* IFN-α production may play an important role in the pathogenesis of HIV-1 infection. However, the effect of IFN is difficult to establish, as many factors may be involved in the implacable immune system activation and dysfunction underlying AIDS progression. Alterations in the production levels of IFN-I were among the earliest reported laboratory abnormalities in AIDS patients, with descriptions of elevated serum levels of an unusual acid-labile IFN-α in patients with Kaposi’s sarcoma and hemophilia [43].

During early infection steps in HIV-infected persons IFN-α production has been reported to be decreased. This process is accompanied by a decrease in the CD4+ T-cell population, and may be used as an additional marker permitting to determine the HIV stage. Contrariwise, higher concentrations of serum IFN-α have been detected in patients with more advanced HIV infection [116, 141] and have been found to correlate with p24 antigenemia [140]. In disagreement with these data it has been reported that asymptomatic non-progressors naturally infected by HIV have augmented levels of IFN-α [132] which correlate with higher levels of CD4+ T-cells, low HIV load and absence of opportunistic infections [44, 47, 132]. In these cases, high levels of IFN-α may play a protective role in HIV/AIDS progression. Similar findings have been reported for HTLV-I and Friend murine leukemia virus (F-MuLV) infections. Viral expression in HTLV-I-infected cells is significantly suppressed when the infected cells are intraperitoneally injected into wild-type mice, but not when they are inoculated into interferon regulatory factor (IRF)-7 knockout mice that are deficient of type-I interferon responses [79]. In mice infected with and treated early during infection with IFN-I, viral loads in spleen and plasma were significantly lower than in untreated mice [52], suggesting that IFN is able to control both HTLV and F-MuLV infections.

The decreased efficacy of IFN with time in controlling retroviral infection may be due to several factors, being one of them the development of IFN-resistant retrovirus clones. Studies on sensitivity to IFN-I have shown that primary HIV isolates derived from patients at various stages of HIV infection displayed a broad range of sensitivity to IFN-α2. The prevalence of IFN-α2 resistance is low in isolates from asymptomatic patients but increases notably once HIV infection progressed to AIDS, when serum levels of IFN-α are higher, and thus it appears that IFN-α either promotes resistance, or favors survival of IFN-α resistant variants [74, 83]. The pathogenesis staging proposed has been that IFN-I synthesis is normal during HIV infection stages I and II, stages when HIV infection is inhibited. However, during the persistent HIV infection, the CD4+ T-cells numbers drop drastically, concurrently with local IFN production. These events are usually followed by infection acceleration, antigenemia and viremia, as well as opportunistic infections which cause the increased and prolonged IFN-I synthesis, which, in turn, leads to IFN tolerance and resistance [74, 81].

In addition, it has been reported that IFN may increase the expression of MHC type I molecules in most T-cells [76], which may originate the selection of CD8+ T-cells [77]. This sustains the hypothesis that high concentrations of IFN-α may be deleterious through the stimulation of cytokine production; activation of pDCs by HIV stimulates IFN-I secretion, which contributes to T-cell activation. T-cells thus activated show reduced proliferation after TCR stimulation [18]. T-cells have also been reported to be affected in HTLV-associated myelopathy/tropical spastic paraparesis (HAM/TSP) IFN-treated patients; the frequency of perforin expression in CD8+ T-cells was significantly decreased after treatment in HTLV-infected patients who experienced clinical improvement, while it was increased in patients who did not experience such an improvement [117]. Thus, the role of type I IFN on retroviral pathogenesis (whether it is protective or harmful) is still unclear.

In SIV the mechanism seems to be similar to HIV, though some differences have been observed. SIV infection may be very virulent/pathogenic for certain species, such as rhesus macaques (*Macaca mulatta*), while for others, which constitute the natural reservoir for SIV, such as sooty mangabeys (*Cercocebus atys*), the clinical outcome does not progress to AIDS and maintain healthy populations of CD4+ T-cells, despite high viral loads [90]. It has been shown that sooty mangabeys have substantially reduced levels of innate immune system activation *in vivo* during acute and chronic SIV infection and their pDCs produce markedly less IFN-α in response to SIV and other TLR7 and 9 ligands *ex vivo* [38, 90, 140]. Thus, the chronic stimulation of pDCs by retroviruses in non-natural hosts may overactivate the immune system and induce the dysfunction underlying AIDS progression, a phenomenon that does not happen in sooty mangabeys, the natural hosts [90, 140].

The low level of T-cell activation in African Green Monkey (*Cercopithecus aethiops*), which is associated with protection against AIDS in nonpathogenic SIVagm infection, may be partly due also to the lack of inflammatory cytokine responses which happens in this species. In SIVagm, bioactive IFN-α was detected in plasma concomitant with the peak of viremia, unlike IL-6 and IL-12 [38]. Thus, in the nonpathogenic SIVagm model, the low level of inflammatory cytokines may be responsible for the low level of T-cell activation, associated with protection against AIDS [38].

In summary, it appears that type I IFN is effective in controlling the *in vivo* infection by retroviruses, though its continual presence may also over-stimulate the immune system to the point where it might be deleterious, both by activating cellular function and apoptosis (see below), and by selecting IFN-resistant clones.

### 3.2. Local IFN levels and their association with dementia

A possible outcome of the infection by some retroviruses is the central nervous system (CNS) involvement, with the development of different degree of dementia. It has been observed that IFN-α levels are increased in the cerebrospinal fluid (CSF) of HIV associated dementia, suggesting that this cytokine may play a role in the pathogenesis of this disease [113, 121]. In a model using mice inoculated intracerebrally with HIV-infected macrophages it was seen that increased levels of IFN-α in the brain correlated directly with cognitive deficits [120]. HIV encephalitis developed in these mice, and blocking IFN-α with neutralizing antibodies significantly improved cognitive function, decreased microgliosis and prevented loss of dendritic arborization in the brains. This effect is also observed *in vitro* [123]. Similar observations correspond to SIV infection, as early antiretroviral treatment is seen to lower brain levels of IFN-α and prevent CNS dysfunction by decreasing brain viral load [94]. Some data suggest that HIV-1 activates pro-inflammatory and IFN-α-inducible genes in human brain endothelial cells and that these genes are linked to the JAK/STAT pathway. According to these data, STAT1 activation plays a crucial role in HIV-1-induced brain damage and in neuropathogenesis [23].

### 3.3. pDCs impairment

An important role of type I IFN in the innate response to HIV-1 infection is suggested by the observation that deletion of pDCs results in rapid progression of HIV-1 infection *in vivo* [86]. In acute and chronic HIV-1 infection, pDC counts [5, 25, 39, 44, 47, 73, 104, 122, 132, 133] and function [5, 14, 25, 40, 44, 47, 73, 133] are severely reduced, reflecting the clinical status of the infected patients and predicting immunological control of HIV-1 replication [105]. Except for the development of dementia, it appears that high levels of IFN-α are beneficial (at least initially, till the immune system gets over-stimulated) and suppress the advancement of symptoms in HIV patients. High HIV proviral loads usually correlate with loss of circulating pDCs, the cell type mostly responsible for the production of IFN-I in response to viruses, and occurrence of opportunistic infections [133].

Why do pDCs counts decrease during some retroviral infections? The decrease of pDCs in blood may not represent an absolute loss of these cells, but rather that they are recruited to specific tissues. To this regard, pDCs are seen to accumulate in the spleen in some HIV-positive patients or migrate to lymphoid tissues [62], possibly driven by p17(MA). This viral protein has been seen to induce immature circulating pDCs to home in lymph nodes, devoid of their ability to serve as a link between innate and adaptive immune systems [48].

A similar phenomenon (declines of blood pDCs numbers but increased presence of these cells in lymph node during acute infection) is also observed in SIVagm infection [38]. pDCs recruited to the lymph nodes display increased, but transient IFN-α production,

pDCs isolated from HIV-1 infected patients demonstrate functional abnormalities, including diminished production of IFN-α when exposed to viruses *in vitro* [87, 141]. Why are pDCs impaired? It has been shown that HIV can directly infect pDCs *in vitro*, providing a potential explanation for their *in vivo* depletion [133, 148]. Synthetic viral Vpr, a weak transcriptional activator of HIV, substantially inhibits IFN-I production by pDCs without inducing their apoptosis [66], supporting the hypothesis that some HIV proteins may affect IFN production by pDCs. In fact, pDCs do not even need to be infected by HIV to show altered IFN production, but are elicited by interactions with HIV-1 virions or certain viral components [148], and the attachment of HIV-1 to pDCs by CD4 through gp120(SU) is critical for subsequent IFN-α production [59]. Nevertheless, diminished production of IFN-I *in vitro* by pDCs from HIV-1 infected patients may not represent diminished IFN production *in vivo*. Thus, the elevations in serum IFN-α levels observed in patients with early infection and advanced HIV disease [141], despite the diminished *in vitro* ability of pDCs to produce IFN-α following stimulation described above have been explained in the sense that IFN-I or even HIV virions may have activated pDCs previously, hindering their IFN-I production when they are later stimulated *in vitro* [141].

In other retrovirus such as HTLV-I, infection of pDCs resulted in impaired IFN-α production and correlated with elevated HTLV-I proviral load in infected individuals [63].

## 4. Effects of Interferon on Retroviral Virogenesis

The *in vitro* effects of IFN-I on retroviruses have been extensively studied. Though most results suggest that IFN affects the latter stages of the viral cycle, important discrepancies are observed. These may be due to different factors, such as whether cell lines (lymphoblastoid *vs* monocytic) or primary cultures (PBMC, monocyte/macrophage lineage, or CD4+ T-cells) are studied, or whether it is an acute or chronic cellular infection. The degree of *in vitro* inhibition may be due to the virus isolate, the target cell, the concentration of IFN, the multiplicity of infection, and the time of infection or of treatment.

Most studies seem to indicate that the effects of IFN-I are more evident when cells are pretreated with this cytokine before viral infection. In studies with HIV, the inhibitory effect of IFN has been observed even when it was added at the moment of infection, since cells (monocytes or T-cells) treated with IFN at the time of virus challenge showed no p24 antigen or RT activity, no HIV-specific mRNA, and no proviral DNA [4, 51, 81, 97, 108, 127]. Contrariwise, it was shown that crude human IFN-α (HuIFN-α) inhibits the replication of FeLV (as evidenced by the titration of the infectious progeny) in feline cell cultures more markedly when it was added within one day after the inoculation of the cells rather than when it was applied before cell infection [69]. In any case, most of the studies have been done treating cells with IFN after retroviral infection.

Parameters studied have been mostly viral protein synthesis and infectivity, though proviral integration has commonly also been determined. Viral protein synthesis is measured either by evaluating the mRNAs or directly measuring the amount of specific proteins. In some retroviruses, such as FeLV, capsid protein is used to detect viremia in infected individuals. It is produced in excess by infected cells, and it may be secreted into the extracellular environment not bound to infectious particles [70]. Infectivity is generally estimated through RT activity in the supernatant of treated infected cells [101], as infective viral particles must carry RT [28]. Infectivity can be also evaluated through syncytia formation [71, 102].

The infectivity of viral particles in IFN-treated cell supernatants was shown to be slightly [51, 131] to drastically [28, 57, 36, 89, 102] reduced. Most studies have shown that protein synthesis is not affected by IFN-I. HIV p24 antigen levels have been reported to remain unaltered in the supernatant after treating infected cells with IFN-I [27, 51, 131]. Other results with HIV showed that the cell-associated fraction of p24(CA) was dose-dependently enhanced by IFN, while the shed fraction of this protein was unmodified [36], and levels of p24(CA) antigen and RT activity in lysates of IFN-treated infected cells were threefold greater than those of controls. In the case of FeLV [28, 147] and FIV [29], levels of capsid proteins in the supernatant of infected cells were not affected by IFN treatment, while RT activity decreased in a dose-dependent manner. These results seem to suggest that IFN interrupts the last stages of the virogenesis, avoiding the correct assembly and the release of viral particles; electron microscopy results support this hypothesis, as it showed that aberrant particles were formed in IFN-treated HIV-infected cells [36].

The effect on protein synthesis may be selective. In the case of HTLV-I, where virus transmission and production were strongly suppressed in the presence of IFN-α or IFN-β, the expression of certain virus specific proteins, such as gp46(SU) (but not p19(MA), p24(CA), p28, p36 and gp68) was affected by IFNs [55, 102], a phenomenon that was not observed by other researchers [46].

Electron microscope reveals that some of the HTLV-I particles in cells treated with high doses of IFN-I are trapped in the intracellular vacuoles [102]. Similar results were observed with some murine retroviruses (Moloney murine leukemia virus, MoMLV), which exhibited altered release of virus when cells were treated with IFN-α, resulting in the accumulation of intracellular virions in cytoplasmic vacuoles [3, 131]. Extracellular RT activity and p24 levels decreased in parallel with increasing IFN, whereas the intracellular viral proteins decreased only slightly [131]. Virions released from these cells contained typical viral RNA, proteins and glycoproteins, as determined by gel-electrophoresis [3, 131]. However, radioimmunoprecipitation analyses of IFN-treated HIV T-cell lysates show that, although viral proteins accumulate in intracellular compartments, they fail to form viral particles. Taken together, the results mentioned above suggest that type I IFNs do not inhibit HIV, HTLV, FeLV, FIV or MoMLV gene expression strikingly, but suppress processing or assembly of virus proteins and/or release of virions in the late phase of maturation.

The mechanism by which IFN may inhibit the assembly and/or release of mature viral particles has also been intensely studied. According to some studies, HIV viral particles from IFN-treated cultures contained a significant lower quantity of the specific gp120(SU), and a decreased ability to bind with their target CD4+ T-cells; gp120(SU) accumulated in aberrant cell compartments, and exhibited altered folding. Thus the diminished infectivity of newly formed particles could be due to defective virion formation [36, 57]. Similar results had been obtained previously for MoMLV, where it was shown that IFN exerted a significant inhibitory effect on the glycosylation of the Env proteins, which may lead to the production of defective noninfectious virions [2].

However, most recent observations suggest that IFN may affect virogenesis by inducing changes on the *gag* gene products [45, 46, 103]. It has been reported that IFN-I may produce a change in post-translation modifications of Gag proteins, as more basic forms of p55, p39 and p24 were detected in HIV-infected stable cell lines, which inhibit capsid polyprotein processing. In addition, particles remaining attached to the cell failed to mature into structures with condensed cores [8]. Other studies suggest that IFN-α blocks HIV-I release mediated by the ubiquitin ligase, ISG-15, one the IFN-induced proteins of the ubiquitin-like pathway. This inhibition occurs by altering protein interaction and ubiquitination steps of Gag required for virus release, without having any effect on the synthesis of HIV-1 proteins in the cell, although the precise mechanism is still unclear [103].

The proper assembly and budding of retrovirus particles are dependent on the interaction of viral Gag polyprotein with lipid rafts at the plasma membrane. Lipid rafts are the dynamic assemblies of selected proteins, cholesterol and sphingolipids that exist in the exoplasmic part of cellular bilayer membranes [21, 88]. Studies with HTLV-I show that IFN-α prevents Gag proteins from associating with the lipid rafts, affecting assembly and thus blocking viral replication [46]. It is not clear whether this alteration is due to the modification of the lipid or proteic composition of the rafts, to its action on the chaperones, to the disturbance of the lipid raft environment critical for IFN-I signaling to STAT proteins, or to post-translational modification of the Gag proteins [45, 46, 93].

Though there is compelling evidence that type I IFNs affect the terminal stages of the retroviral cycle, other studies seem to prove that IFN may inhibit the early phases of replication, decreasing virus uptake and entry, DNA synthesis by RT, the levels of integrated provirus and of RNA and proteins expressed [9, 36, 126, 127]. These discrepancies may be due to the retrovirus infection status of the cell; in *de novo* infections (*i.e.* cells which are infected for the experiment), the effect of IFN-I may be in the early stages of the cycle, while in the chronic infections (primary cells or cell lines already infected), IFN may inhibit replication in the later stages [36, 126]. It was reported that IFN-α inhibited effectively the first HIV-1 replication cycle by decreasing the relative RNA and protein levels in cells. The levels of integrated proviral DNA were significantly lower in IFN-α treated cells compared to non-treated controls, suggesting that the effect of IFN-α was at the level of proviral DNA formation or integration [127].

The discrepancies might be associated also to specific retroviruses [126]. To this respect, viral specific mRNAs have been shown to decrease markedly in SIV-infected IFN-treated cells, but not in HIV-1-infected cells. It was concluded that IFN inhibited an earlier stage of replication in SIV-infected cells, in a step between attachment and reverse transcription [137].

Several mechanisms involving genes induced by IFN have also been proposed. The expression of the genes encoding for Mx and OAS induced by IFN-I has been reported to inhibit the formation of viral particles and to reduce HIV-1 RT activity [9, 126] and p24 synthesis [131].

Over the past few years some intracellular proteins with antiretroviral activities, referred as restriction factors, have been described in several infected host cells. Examples of these molecules are APOBEC3G/F and TRIMs families. APOBEC3G/F are cytidine deaminases that are able to strongly inhibit retroviral replication and that are neutralized by lentiviral Vif protein. TRIM5α may block reverse transcription by rapidly recruiting TRIM5α-associated virus to the proteosome and degrading it (reviewed in 67, 84], although very often TRIM from a particular mammalian species does not restrict infections by retroviruses of the same mammalian species. Both antiretroviral factors, APOBEC3G/F and TRIMs, are upregulated by IFN-α in infected cells, resulting in enhanced antiviral activity against HIV-2, SIVmac, or N-tropic MuLV infection. Moreover, high expression levels of TRIM5α play an essential role in controlling both the initial retroviral exposure and the subsequent viral dissemination *in vivo* [118]. Recently, the ability of TRIMs to block retroviral replication in the late stages of viral particle production has been described in HIV-1 infected cells, which appears to account for some of the interferon’s antiviral activity against HIV-1 budding [67].

## 5. Effects of IFN at the Cellular Level: Apoptosis

In the preceding section, it has been discussed that type I IFNs inhibit the final release of retrovirus particles from the cells, resulting in an accumulation of cell-associated virions, or producing defective noninfectious virus particles, apparently due to the interference with the processing of viral proteins and their assembly into complete virions, without altering significantly retroviral protein synthesis. This prompted researchers to hypothesize that these effects of IFN-I are a result of a cellular interaction with IFN rather than a result of the antiviral activity of IFN [32].

One of the effects of IFN on the cell is the induction of apoptosis. Apoptosis plays a critical role in cellular differentiation, in the elimination of cells that sustain genetic damage or undergo uncontrolled cellular proliferation, and in preventing viral replication by eliminating virus-infected cells [24]. The potential of IFN to induce apoptosis has been known for some time. In general, the prolonged activity of IFN-induced proteins leads to cell death by apoptosis, a response that certainly limits spread of virus from one cell to another. IFN activates the expression of genes which contribute to apoptosis. For example, STAT1, which is critical for signaling for both types of IFN, has been proposed to play a role also in apoptosis. PKR molecules regulate the expression of genes involved in apoptosis, possibly involving signaling through the NF-κB pathway [13]. Though IFNs are positive mediators of cell death, there are instances where both type-I and –II IFNs may actually prevent apoptosis [13].

Strong and sustained induction of TRAIL and/or Fas/FasL in response to IFN has been shown to lead to recruitment and activation of the Fas associated death domain (FADD). FADD plays an important role in IFN mediated apoptosis as transfection experiments, using dominant negative mutants of FADD, conferred IFN-resistance to sensitive cells. FADD activation, in turn, activates caspase-8, initiating activation of the caspase cascade. Activated caspase-8 cleaves Bid, a proapoptotic member of Bcl2 family, resulting in disruption of mitochondrial potential, release of cytochome *c* from the mitochondria into the cytoplasm, where it acts as a cofactor to stimulate the complexing of Apaf1 with caspase-9. This complex potentiates caspase-3 activation, followed by changes in plasma membrane symmetry, cleavage of PARP, chromatin condensation and DNA fragmentation and cell death [24].

It was long envisioned that the effect of IFN on retroviruses could be a consequence of its interaction with the cell membrane. Thus, apoptosis induction and the existence of lipid rafts were predicted [2]. Using selective stains, type I IFNs have been shown to induce apoptosis in FeLV- and FIV-infected cells, to a degree several orders of magnitude higher than in non-treated cells [28, 29]. It was concluded that the intracellular and/or the slight changes in the cell membrane derived from the synergic action of viral infection and IFN-I could lead to a reduced release of viral particles (evaluated indirectly by the RT activity). This would limit the spread of the infection to other cells, and originate the selective death of infective cells [28].

The effects of apoptosis induced by IFN are not limited to retrovirus-infected cells. More notably apoptosis stimulated by IFN-I has been proposed as a mechanism to explain non-infected bystander CD4+ T-cells depletion and disease progression in HIV infection [1, 60, 61, 68, 92, 96]: *in vitro*, HIV-1 gp120(SU) in the membrane of infected CD4+ T-cells stimulates the production of IFN in dendritic cells, which induces the expression of TRAIL by infected and non-infected CD4+ T-cells [61]. According to this model, in progressing individuals with high plasma viral loads, HIV binds to CD4 on pDCs, resulting in their activation, IFN-α production and migration from blood to lymphoid tissues. IFN-α binds to its receptor on infected and non infected CD4+ T-cells (resulting in STAT-1/2 regulated expression of membrane TRAIL), and HIV gp120(SU) binds to CD4 on these cells. This latter event is required for the expression of the TRAIL death receptor 5 (DR5). Given that a high percentage of viral particles are not infective, but they still carry gp120(SU), DR5 is expressed on many non-infected CD4+ T-cells. TRAIL binds to DR5, resulting in CD4+ T-cell apoptosis. Contrariwise, in non progressing patients with very low plasma viral loads, pDCs do not produce IFN-α and the expression of TRAIL is not induced, and the previous cascade, resulting in apoptosis, is not initiated [60]. According to another hypothesis, the viral protein Nef, a virulence factor that plays multiple roles in HIV replication, is capable of inducing proapoptotic effects in uninfected bystander cells, and antiapoptotic effects in infected cells. Nef activates the synthesis and secretion of a set of chemokines/cytokines that activate STAT1 and STAT3, as well as IRF-3, leading to the synthesis of IFN-β, which, in turn, induces STAT2 phosphorylation [92].

This cytotoxic mechanism and the stimulation of NK cells [145] may be responsible for the high degree of apoptosis observed in non-infected CD4+ T-cells in HIV-1 infected patients [42]. Other mechanisms involve the upregulation by HIV-1 of the gene expression of p53, associated with HIV-mediated IFN-I synthesis [68], or the induction of IRF-1 by HIV-1, as this factor is clearly involved in apoptosis of activated T-cells and in the context of the machinery that T- cells use to induce apoptosis in their target cells [96].

## 6. Direct Effects of Type I Interferons at the Molecular Level: ISREs

As mentioned previously, ISREs are DNA sequences which bind with the well-conserved N-terminal region of the interferon regulatory factors (IRFs). By this mechanism, IRFs regulate the expression of genes stimulated by IFN, activating or either repressing gene transcription, depending on the target gene. IRFs are involved in multiple biological processes including regulation of immune responses and host defense, cytokine signaling, cell growth regulation and hematopoietic development. ISREs are present on the promoters of the target genes, *i.e.* the IFN-α or IFN-β genes and some IFN-stimulated genes (ISG) (for a review of different ISRE sequences, see 142]. Sequences homologous to the ISREs have been identified in several retroviruses. For example, in HIV a sequence (TTGAAAGCGAAAGGGAAACC) homologous to ISRE has been identified in the 5′ HIV-1 LTR downstream of the HIV-1 transcription start site (Figure 1). This sequence is a binding site for several IRFs and its deletion results in impaired LTR promoter activity and altered synthesis of viral RNA and proteins [15]. *In vivo* it recruits IRF-1 and IRF-3 [144]. Other researchers have reported that it has a binding site for IRF-1 and IRF-2 [15, 96], but only IRF-1 is able to stimulate HIV-LTR transcription (and reactivates provirus from latency), interacts with Tat [124], and increases HIV-1 replication [96, 125]. HIV is able to induce IRF-1 early upon infection, before the expression of Tat, and it is possible that it plays an important role in the early phases of HIV infection and as a strategy to counteract IFN-mediated host defenses [124, review in 125] On the other hand, IRF-8 represses IRF-1-Tat-mediated transactivation of the LTR by interfering with IRF-1-Tat association, and it inhibits HIV-1 replication in CD4+ cells [96, 99].

In FeLV the sequence GGTTTCATTTTCG, matching the consensus NAGTTTCNNTTTCNC/T [146], was found at nucleotide 798, in a region just upstream from that which encodes the Gag-Pol precursor polyprotein gPr80; however, as it is not in the LTR regulatory region, it is difficult to link it with the initiation of transcription of the viral genome [28].

The region situated immediately downstream from the transcription start site (U5) in the BLV LTR is involved in regulation of viral gene expression. It contains a positive regulatory element in its 5′ region (nt +230 to +275) that functions either at the transcriptional or past-transcriptional level. This U5 region contains a sequence (nt +251 to +261) highly similar to an ISRE (TACTTTCTGTTTCTCG), which binds IRF-1 and IRF-2, but not ISGF3, and thus can be considered an IRF binding site, rather than a classical ISRE. Its failure to confer IFN responsiveness is most probably due to its inability to bind ISGF3. This motif is required for optimal basal gene expression from the BLV LTR [78].

Up till now, no ISRE sequences have been identified in HTLV, FIV or other retroviruses, despite the effects of IFN on these viruses, which suggests that the molecular mechanisms involved may be more complex than anticipated and are still to be clarified.

## 7. Mechanisms by which Retroviruses May Evade the Effects of Type I Inteferons

**Figure 5.**
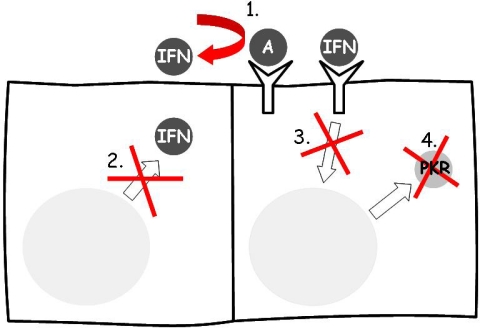
Mechanisms by which viruses circumvent the effect of IFN. 1. Competition with IFN for receptors (IFNAR); 2. Inhibition of IFN synthesis and secretion; 3. Inhibition of the IFN signaling, which may happen at different levels; 4. Inhibition of the proteins stimulated by IFN, mostly of the PKR system.

Viruses have evolved different mechanisms that allow them to evade the antiviral response induced by IFN. This anti-IFN activity may be produced at three distinct time points: during IFN synthesis, during signaling, and through the alteration of the antiviral proteins induced by IFN. Some viral proteins are suppressors of IFN gene expression through their general inhibitory effect on host gene transcription [56]. However, they can also evade specifically the action of IFN. The major strategies include [45, 112] (a) competition for binding to IFN receptors; (b) inhibition of IFN production and secretion; (c) blockage of the signal of IFN; and (d) inhibition of the antiviral proteins induced by IFN or of their actions (Figure 5).

The mechanisms by which retroviruses evade IFN control belong to the fourth category. The presence of increasing levels of IFN in the serum of AIDS patients while viral replication continues and the disease progresses indicates that HIV-1 must employ a mechanism to evade the antiviral effects of IFN. However, the complexity of the virus-host interactions and the profound disregulation of the host cytokine network exerted by the virus at different stages during the infection have made these studies extremely difficult. In response to viral infection, IFN induces a number of genes including the dsRNA-dependent protein kinase R (PKR). PKR exerts its anti-viral activity by phosphorylating the alpha subunit of eukaryotic translation initiation factor-2 (eIF-2), which results in the shut-down of protein synthesis in the cell. However, PKR activity is inhibited directly by HIV via the major regulatory protein, Tat [41]. This protein, in association with cellular factors, enhances the efficiency of transcription by cellular RNA polymerase 1000-fold, mainly by preventing premature termination of transcription. It triggers efficient RNA chain elongation by binding to TAR RNA, which forms the initial portion of the HIV-1 transcript. HIV-1 TAR is a highly-conserved stable RNA stem loop that interacts with Tat protein to regulate viral transcription [19]. HIV-1 Tat protein has been shown to act also as a substrate homologue of eIF-2, competing with it and preventing the phosphorylation of this factor, allowing protein synthesis and viral replication to proceed in the cell. Tat protein reduces the activity PKR significantly, while TAR RNA blocks its activation [41].

Nef, a crucial determinant for HIV replication and pathogenesis, is believed also to play a major role in the evasion of the virus from the effect of IFN, by manipulating the phenotypical, morphological and functional characteristics of pDCs, rendering them incapable of activating CD8+ T-cells and down-regulating their proliferation and functional competence [110].

The involvement of IRFs in HIV-1 replication allows to speculate that targeting IRFs (IRF-1, IRF-2 and IRF-8) can be also regarded as a mechanism utilized by HIV-1 to evade the antiviral effect of IFN [96]. Other proteins of HIV degrade PKR and induce an inhibitor of RNaseL [96, 128].

HTLV blunts the expression of some genes induced by IFN, probably depressing the MxA, PKR and OAS pathways. Recently it has been described that HTLV-I did not have an effect either on the initiation of the IFN process (as it did not affect the cell surface presentation of IFNAR1 and IFNAR2 or IFN-α binding). However, it reduced the phosphorylation of TYK2 and STAT2, and to a lesser extent, of JAK1 and STAT1, suggesting that the virus inhibits the IFN-induced specific activation of these cellular proteins [45]. Evidence that Gag and Pro may be responsible for this HTLV-I mediated IFN-α inhibition has also been provided [45]. These effects have also been ascribed to the viral protein Tax, a pleiotropic transcription factor that interferes with several of the cellular mechanisms and modulates transcription of a wide range of cellular genes [20]. It has been proposed that Tax protein of HTLV-I acts as an IFN-α antagonist, as it negatively modulates IFN-α induced JAK-STAT pathway by competing with STAT2 for coactivator CBP/p300, thereby inhibiting the transcription activation of STAT2-containing ISGF3 complex [150]. Tax may prevent by this mechanism IFN-α from exerting its antiviral, antiproliferative and proapoptototic effects, contributing to persistent viral infection and HTLV-I associated oncogenesis [150]. Nevertheless, it is possible that different HTLV-I gene products, other than Tax, may act at different levels of the IFN signaling pathways, coexisting more than one mechanism to evade IFN action, as has been shown for other viruses.

## 8. Other Type I Interferons

### 8.1. Interferon-tau (IFN-τ)

IFN-τ is a noncytotoxic type I IFN responsible for maternal recognition of pregnancy in ruminants. It has been shown to have potent antiviral and antiproliferative activity. Data suggest that its anti-HIV activity is higher than IFN-α/β on primary PBMC and monocyte-derived macrophages infected *in vitro* by HIV [35]. Also, it induces IL-6 and IL-10 synthesis [91, 115]. The IFN-τ antiretroviral activity is not associated with a decrease in either cell viability or immune reactivity [109], supporting the interest for the IFN-τ as an adjuvant therapy drug in HIV infection [26].

Like other IFNs, it seems to affect several steps of the HIV replication cycle. IFN-τ effectively inhibits the early steps of the biological cycle of HIV replication, particularly in human monocyte-derived macrophages, decreasing intracellular HIV RNA and inhibiting the initiation of the reverse transcription of viral RNA into proviral DNA. Anti-HIV effects of IFN-τ are mediated by several modes of action, either directly by IFN-τ, or via other cytokines [91]. The mechanisms proposed are i) synthesis of cellular antiviral factors such as 2′,5′-OAS/RNase L and MxA protein [26], and ii) increased production of MIP-1α, MIP-1β, RANTES, natural ligands of CCR5, the principal coreceptor of HIV in macrophages [114].

The effects of IFN-τ have also been studied on BLV. BLV titers decreased in BLV-infected cells (FLK-BLV) and in peripheral blood mononuclear cells of BLV-infected cattle treated with recombinant bovine IFN-τ (rBoIFN-τ), demonstrating that this cytokine could directly inhibit BLV propagation rather than acting through its immunomodulatory effects [80].

Significant dose-dependent inhibition of reverse transcriptase activity by IFN-τ was detected by day 6 of culture in FIV-infected feline PBL treated with IFN-τ. In addition, the production of the FIV core protein, p25(CA), was blocked. Both the amino- and carboxyl-terminal regions of IFN-τ, as identified by synthetic peptides, appeared to be involved in its antiretroviral activity. IFN-τ antiretroviral activity was not associated with a decrease in either cell viability or immunologic reactivity [109].

IFN-τ has an effect on the immune response of sheep. The immunomodulatory role of recombinant ovine IFN-τ (rOvIFN-τ) included the increase of the proportions of primary antiviral γδ^+^and CD8+ immune cells in ovine lentivirus (SRLV)-infected lambs. This may represent a cellular mechanism to explain the antiviral and therapeutic efficacy of this cytokine, in addition to its direct antiviral effect [129]. The effect of rOvIFN-τ on the replication of SRLV in goat synovial membrane cells was studied by Juste *et al.* [71]. The strongest inhibitory effects were on syncytia formation and release of infectious virions into the cell culture supernatant, though the production of RT was not significantly different in cells treated with IFN-τ and in control cells. These results suggest that, as other type I IFNs, the action of this IFN is mostly on the latter steps of SRLV replication cycle, possibly by blocking virus assembly and/or release, thus limiting the spread of the infection [71]. *In vivo* results demonstrated a 90% reduction in SRLV titres in lambs infected experimentally with SRLV and that received early rOvIFN-τ treatment [72].

### 8.2. Interferon-omega (IFN-ω)

IFN-ω is also a potent inhibitor of HIV replication *in vitro*; both laboratory and primary isolates of HIV-1 are more sensitive to IFN-ω than to IFN-α2, and protein synthesis is inhibited by IFN-ω to a greater degree than by IFN-α2. Data suggest that the expression of ISGs, particularly that of ISG-15, is higher and more sustained on treatment with the former than with the latter, which may confer a higher therapeutic index to IFN-ω in controlling HIV infection [82].

The *in vitro* effect of commercially available recombinant feline interferon omega (rFeIFN-ω) has been evaluated on the expression and replication of FeLV [28] and FIV [29, 136]. Very similarly to IFN-α, rFeIFN-ω induced a marked inhibition of RT activity (and thus of infectivity), whereas it had no effect on protein synthesis.

In summary, type I IFNs seem to play an important role in the innate immune response against retroviruses, but their effect is not well established. *In vivo* IFN-I levels vary throughout retroviral infections, though findings are contradictory, as for some researchers, high levels seem to be beneficial for the host to fight infection, while for others, they would provide a mechanism for deleting by apoptosis non-infected bystander cells. However, exogenous treatment with IFN-I appear to be generally valuable. *In vitro* findings support that type I IFNs undoubtedly inhibit replication of retroviruses, though there is not an unanimous agreement about whether it is at the early or late stages of virogenesis, and of whether it is by directly affecting the replication process or by altering cellular viability through apoptosis. Some retroviruses have ISRE-like sequences, which might modulate viral expression, and some are able to evade the effects of IFN, possibly through the regulatory proteins.
